# Developing a mechanism-based therapy for acute psychiatric inpatients with psychotic symptoms: an Intervention Mapping approach

**DOI:** 10.3389/fpsyt.2023.1160075

**Published:** 2023-06-01

**Authors:** Eva Gussmann, Susanne Lucae, Peter Falkai, Frank Padberg, Samy Egli, Johannes Kopf-Beck

**Affiliations:** ^1^Max Planck Institute of Psychiatry, Munich, Germany; ^2^Department of Psychiatry and Psychotherapy, LMU University Hospital Munich, Munich, Germany; ^3^Department of Psychology, LMU Munich, Munich, Germany

**Keywords:** intervention mapping, intervention development, mechanism-based, acute inpatients, psychosis, metacognition, group therapy

## Abstract

**Background:**

Treatment guidelines for psychosis recommend offering psychotherapy already in the acute illness phase. However, there is a lack of available interventions adapted to the specific needs and key change mechanisms of inpatients experiencing severe symptoms and crisis. In this article we outline the scientific development process of a needs-oriented and mechanism-based group intervention for acute psychiatric inpatients with psychosis (MEBASp).

**Methods:**

To guide our intervention design, we used Intervention Mapping (IM), a six-step framework for developing evidence-based health interventions that consisted of an extensive literature review, an in-depth problem definition and needs analysis, the modeling of change mechanisms and outcomes and the production of an intervention prototype.

**Results:**

Our low-threshold modularized group intervention consists of nine stand-alone sessions (two per week) within three modules and targets different aspects of metacognitive and social change mechanisms. Module I and II aim to reduce acute symptoms by fostering cognitive insight, Module III focuses on reducing distress via cognitive defusion. Therapy contents are adapted from existing metacognitive treatments such as the Metacognitive Training and presented in a destigmatizing, simply understandable and experience-oriented way.

**Conclusion:**

MEBASp is currently evaluated in a single-arm feasibility trial. Using a systematic and rigorous development methodology and providing a detailed description of the development steps demonstrated to be invaluable in improving the intervention’s scientific foundation, validity, and replicability for similar research.

## 1. Introduction

Psychological therapies have demonstrated to be effective for patients experiencing psychotic symptoms (1, 2) and are recommended by treatment guidelines already in the acute illness and treatment phase (3, 4). However, recent systematic reviews and meta-analyses investigating treatment effects for acute psychiatric inpatients with psychosis revealed an outcome superiority of third-wave therapies (5–7) over guideline-recommended second-wave cognitive behavioral therapy for psychosis (CBTp) (3, 4). In contrast to disorder-specific CBTp protocols that aim to alter the occurrence and form of psychotic symptoms such as delusional thoughts and hallucinations (8), third-wave therapies often focus on the behavioral function of internal experiences rather than their content per se (9). Instead of examining and disputing the content of voices and thus giving them increased attention and importance for example, third-wave therapies train patients to mindfully experience auditory hallucinations in order to reduce their negative impact on behavior (10). They also emphasize the therapeutic importance of targeting evidence-based change mechanisms, which are the underlying (psychological) processes responsible for positive treatment outcomes, instead of solely focusing on changing symptoms (11). Third-wave interventions e.g., aim at changing impaired reasoning processes behind delusional thoughts and not necessarily the content of the specific delusion (9). Change mechanisms thereby draw on impaired processes believed to contribute to the maintenance and onset of various mental health problems and thus often operate as transdiagnostic change factors (11). Cognitive distortions associated with depressive disorders for instance can also be improved through interventions targeting general reasoning abilities (12). Understanding what leads to change and tailoring therapy to directly address those change mechanisms hence seems to be important to generally optimize therapeutic strategies and thus to improve overall treatment outcomes for patients (9, 13).

Given the urgent need for effective inpatient care (14, 15), prioritizing change mechanisms in therapy therefore might hold a great potential to positively impact disease progression and prognosis of patients with acute psychosis (16). Major third-wave therapies that explicitly focus on potential change mechanisms in psychosis are the Acceptance and Commitment (ACT) and the Metacognitive Training (MCT) (9). ACT for instance fosters acceptance and cognitive distancing from delusions and hallucinations (17) and has shown to reduce general psychopathology and rehospitalization rates in acute inpatients with psychosis (18–20). MCT on the other hand aims to promote patients’ cognitive flexibility by raising metacognitive awareness and knowledge for cognitive biases (21) and showed significant effects on reducing positive symptoms (8, 22, 23). Although the mechanism-based principles of these approaches seem promising in the treatment of acute inpatients with psychosis, existing evidence has to be treated with caution (5). Until now, evidence is based on a small number of randomized controlled trials (RCTs) with relatively heterogeneous study conditions and methodological shortcomings (5–7). On top of that, ACT and MCT were developed for outpatient settings where patients’ symptom severity and hence key change mechanisms and needs can be assumed to differ from those of patients experiencing acute crises (24). Change mechanisms in acute inpatient environments for example mainly comprise of mechanisms associated with distress and risk reduction (16), while outpatient therapy focuses on processes like value commitment that support long term recovery goals (1). In addition, acute psychiatric settings by themselves represent challenging environments to deliver psychotherapy, counting involuntary admissions, brief inpatient stays and staff shortage as major obstacles (25). Researchers therefore argue that further intervention development is needed that (a) identifies and adapts to specific inpatient change mechanisms and (b) reflects the complex requirements of acute psychiatric ward (25–28).

However, the actual development process of interventions in psychotherapy is often kept short and under-reported (29). Neglecting the actual development phase can be problematic, as a poor problem definition, insufficient attention to existing evidence and context needs, a missing model underlying the intervention, and an unsound selection of hypothesized change mechanisms can lead to inefficient treatments (30–32). An “intervention black box” then makes it difficult to understand why specific therapy components didn’t work in a clinical trial (31). Furthermore, a published, in-depth description of the development process is necessary for other researchers to replicate findings and for clinicians to understand how to implement the intervention (33).

In order to overcome these shortcomings, Bleijenberg et al. (31) suggest using structured methodological frameworks such as the Intervention Mapping (IM) that fulfills the Medical Research Council’s (MRC) quality criteria on intervention development (31, 32). Although the use and reporting of IM approaches is prevalent in health and prevention research (34–39), there are only a limited number of comparable academic articles published in the field of (clinical) psychology (40, 41). The current article’s objectives are therefore twofold: We aim to describe the development and theoretical underpinnings of a mechanism-based and needs-oriented intervention for inpatients with psychosis (MEBASp) treated in an acute psychiatric setting. By using Intervention Mapping in doing so, we also hope to provide an example and highlight the benefits of how existing rigorous development frameworks can be used to enhance the design and reporting standards for psychological therapies in psychiatric research.

## 2. Materials and methods

**FIGURE 1:**
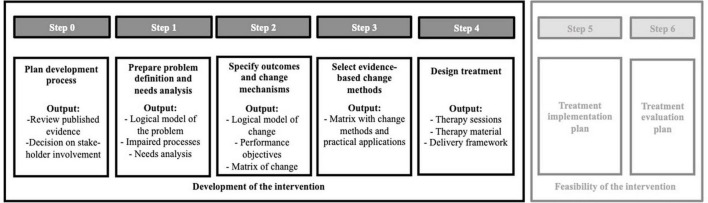
Illustration of the IM intervention development process and selected steps undertaken in the MEBASp project.

We chose IM as our conceptual development framework due to its systematic and detailed protocol allowing an effective selection of treatment mechanisms and procedures in six consecutive steps (42). In the practical application of those steps, we were guided by the approach of van Agteren et al. (40), who adapted the IM method for mental health research. Next to following IM principles, we made sure to adhere to relevant reporting guidelines (e.g., Template for Intervention Description and Replication) when describing and explaining our development milestones (33). Figure 1 provides an overview of the development steps undertaken to design our intervention that are described in detail in the sections below.

### 2.1. Step 0: planning process and decision on stakeholder involvement

Next to theory and evidence-based development principles, the IM approach emphasizes participatory research activities e.g., involving the target population in all planning phases trough qualitative research (43). Collaborative care planning approaches, such as codesign and coproduction, have thereby become increasingly important in mental health intervention design and delivery, and have been shown to improve service quality (44–47). Nevertheless, the implementation of codesign in psychiatric research settings can be challenging due to the significant time and cost involved (48), as well as the ethical challenges that arise when conducting qualitative research with severely burdened and highly vulnerable patient groups (49, 50). To address this challenge, Locock et al. developed an accelerated codesign approach that drew on pre-existing qualitative patient data and that proved to be acceptable to patients and staff (48). Building on this approach, we first of all reviewed pre-existing qualitative research involving acute inpatients with psychosis (for an overview see Supplementary Table 1). Published studies were primarily conducted in a psychiatric context in the UK, which was found to be very similar to the German system (51), thus making available data transferable to our current research context. By deciding to draw on secondary data for our project instead of conducting primary research, we aimed to take advantage of synergistic effects by implementing patients perspective from prior research, while also considering the constraints of time and resources discussed above. However, we included various codesign activities in our subsequent feasibility study such as feedback rounds and questionnaires, and interviews with both participants and staff (see future directions) to ensure that the intervention prototype will be refined according to the needs and preferences of our target population (52).

### 2.2. Step 1: logical model of the problem and needs analysis

**FIGURE 2:**
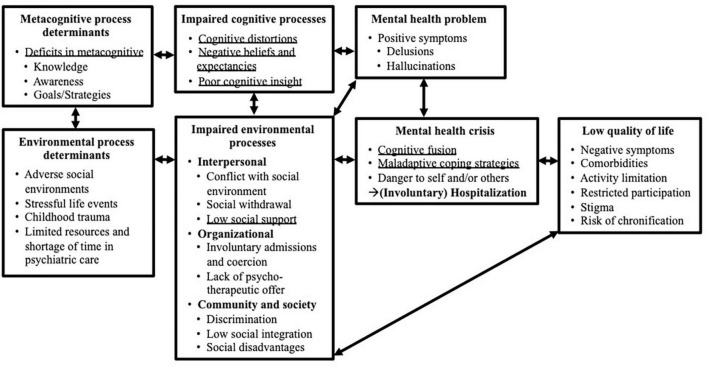
Logical model of the problem of severe psychotic symptoms, danger to self and others, (involuntary) hospitalization and a resulting low quality of life (Step 1). The model has a focus on psychological and social factors in the development of psychosis and does not consider biological factors e.g., genetics. It moreover does not map the moderating or mediating relationships between variables, but rather aims to visualize the variability of factors and impaired processes that contribute to these main health problems (40). Impaired processes that were identified as target areas for the logical model of change are underlined.

The first step of IM involved an exact description of our development context including our target population and setting. We moreover conducted an extensive literature study to create a logical model (theory) of our problem (see Figure 2) from which we derived the theoretical underpinnings, the requirements for and the scope of the intervention (43). To structure the literature research behind the problem determination and resulting needs analysis, IM suggests using the PRECEDE-framework (an acronym for Predisposing, Reinforcing and Enabling Constructs in Educational Diagnosis and Evaluation), which is an established research method to assess health issues on the basis of four predefined assessment phases (53). Going through the different phases, research teams ask themselves the following questions: What is the problem and who has it (epidemiological assessment)? How does it affect patients (social assessment)? What may be its causes (ecological assessment)? How do policies contribute to the problem (policy assessment)? (54). Following the framework’s phases, we covered information on (1) mental health problems of acute inpatients with psychotic symptoms, (2) their effects on quality of life (QoL), (3) potentially associated pathogenetic psychological and environmental processes causing the problem and 4) characteristics (policies) of acute psychiatric wards. Our sources of information included systematic reviews and meta-analyses (5–7, 55), qualitative interview studies (16, 25, 28, 56, 57), core competency frameworks and existing mechanism-based therapies for working with acute inpatients with psychosis (8, 18, 20, 22, 23, 26, 58, 59).

Impaired psychological processes e.g., cognitive distortions found to be relevant in psychosis (60) were grouped into different overarching process domains such as cognition (see Supplementary Table 2). A psychological process thereby refers to an aspect of human cognition, affect, behavior or physical sensation that may be involved in the predisposing, etiology or maintenance of a disorder (61). As impaired processes are believed to causally interrelate with several mental disorders (62), we made sure to include transdiagnostic findings in our overview. To organize the overview, we utilized the available subdivisions found in the transdiagnostic process collection by Harvey et al. (61) which summarizes research results on cross-diagnostic altered processes in five different domains. Using existing process-oriented etiological models for psychosis (63–67), we then identified the most important environmental and psychological processes for our problem model. Existing intervention concepts focusing on identified processes as mechanisms of change were then extensively studied to estimate common practices, their effectiveness and potential barriers (31) (see Supplementary Table 3).

### 2.3. Step 2: intervention outcomes, change mechanisms and logical model of change

**FIGURE 3:**
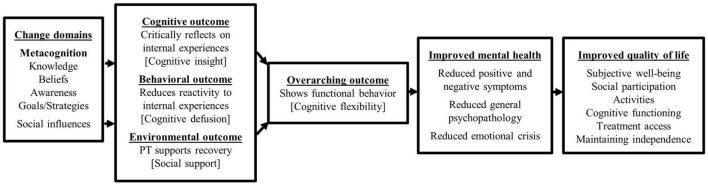
Logical model of change showing what change is needed to manage the main health problems of severe psychotic symptoms and danger to self and others (Step 2). It points out the change domains and belonging change mechanisms expected to influence the cognitive, behavioral and environmental outcomes that are in turn believed to improve mental health and quality of life. Hypothesized underlying target change mechanisms are put into square brackets.

In a second step, we used our logical problem model and needs analysis (Step 1) to define desired cognitive, behavioral and environmental intervention outcomes necessary to prevent or reduce our health problems (e.g., patient critically reflects on internal experiences) and thus positively influence quality of life effects. Following the IM framework, we then addressed the question of *why* patients would make these changes by selecting impaired processes from our problem theory (e.g., poor cognitive insight) and rewriting them into hypothesized change mechanisms (e.g., higher cognitive insight) (43). Overarching change domains were chosen from the Theoretical Domains Framework (TDF) (68), an integrative framework that provides intervention developers with a possible selection of 14 change domains e.g., behavioral regulation and 84 change mechanisms e.g., self-monitoring from evidence-based behavior change theories. We summarized our overall findings in a graphical logical model (theory) of change (43) (see Figure 3).

Our intervention outcomes were further divided into so-called performance objectives (e.g., Patient understands the cognitive model of CBT) (see Table 1). These objectives describe specific behaviors that need to be pursued in order to reach the desired treatment outcome (43). By linking performance objectives with selected change mechanisms from above, we were able to phrase specific change objectives. Simply put, change objectives concretely verbalize what occurs through a change mechanism (e.g., The patient demonstrates increased knowledge about the impact of internal experiences on behavior) (40). As a result, all change objectives were organized in a matrix of change (43) (see Table 2).

**TABLE 1:** Expected cognitive, behavioral and environmental outcomes and performance objectives (PO) for MEBASp (Step 2).

**Cognitive outcome 1: critically reflects on internal experiences**	
PO 1.1.	Understands the cognitive model of CBT
PO 1.2.	Understands the negative consequences of cognitive biases on mental health
PO 1.3.	Considers multicausal explanations for situations and internal experiences
PO 1.4.	Gathers sufficient information before drawing decisions
PO 1.5.	Considers a variety of information when assessing someone
PO 1.6.	Formulates helpful alternatives for depression-inducing thought patterns
PO 1.7.	Knows positive activities to counteract depressed mood and low self-esteem
**Behavioral outcome 2: reduces reactivity to internal experiences**	
PO 2.1.	Understands the negative consequences of fusing with internal experiences (thoughts, delusions and hallucinations)
PO 2.2.	Understands that most internal experiences are produced by the mind and learned in the past
PO 2.3.	Actively perceives internal experiences without directly reacting to them
PO 2.4.	Differentiates between helpful and unhelpful internal experiences
PO 2.5.	Deploys various functional coping strategies in dealing with internal experiences
**Environmental outcome 3: psychological therapy (PT) supports recovery of individual**	
PO 3.1.	PT is accessible for acute patients with psychotic symptoms
PO 3.2.	PT is adapted in scope and complexity for acute patients
PO 3.3.	PT provides social support and enables exchange with fellow patients
PO 3.4.	PT normalizes and destigmatizes mental health problems
PO 3.5.	PT supports patients to apply functional coping strategies in everyday life

**TABLE 2:** Matrix of change for cognitive, behavioral and environmental outcomes showing the change objectives for each performance objective and change domain (Step 2).

	Key change domains			
	Increases knowledge about …	Raises awareness of …	Builds up skills to …	Changes beliefs to …
	[Metacognitive and cognitive knowledge]	[Metacognitive awareness and cognitive attention]	[Behavioral and (meta-) cognitive strategies]	[Metacognitive beliefs]
**Cognitive outcome 1: critically reflects on internal experiences [Cognitive insight]**				
**PO 1.1.**	**K1.1** Influence of thoughts on feelings and behavior	**A1.1** Internal experiences	**S1.1** Report on internal experiences [Introspection]	**B1.1** Behavior is controllable
**PO 1.2.**	**K1.2** Nature of cognitive distortions and their impact on mental health problems	**A1.2** Selective attention/Attentional biases	**S1.2** Anticipate consequences of internal experiences on behavior [Expectancy reasoning]	**B1.2** Thoughts are prone to error
**PO 1.3.**	**K1.3a** Attribution types (internal, external, control possibility)	**A1.3** Attributional biases (Self-serving bias/Pessimistic attributional style)	**S1.3** Rationally analyze events [Attributional reasoning]	**B1.3** Events are always multicausal
	**K1.3b** Dysfunctional attributional styles and their effect on mental health			
**PO 1.4.**	**K1.4a** Rationale behind premature decisions	**A1.4** Jumping to conclusions (Arbitrary inference/Belief bias)	**S1.4a** Gather and process information	**B1.4** Sufficient information is necessary for reasonable conclusions
	**K1.4b** Effect of JTC on mental health		**S1.4b** Actively challenge own conclusions and adjust if necessary [Information processing/ Interpretative reasoning/ Self-reflection]	
**PO 1.5.**	**K1.5a** Rational behind theory of mind	**A1.5** Hasty first impressions (Selective abstraction/Biased expectancy/Availability heuristic)	**S1.5a** Consider contextual information in social interactions	**B1.5** Sufficient information is necessary to assess my opposite
	**K1.5b** Effect of distorted mentalizing on mental health		**S1.5b** Take different perspectives **S1.5c** Tolerate ambiguity [Cognitive shifting/ Interpretative reasoning/ Social reasoning]	
**PO 1.6.**	**K1.6a** Dysfunctional cognitive patterns	**A1.6** Depressive-inducing thinking patterns (Catastrophizing/Personalization/Over-generalization)	**S1.6** Come up with functional thoughts [Cognitive reappraisal]	**B1.6** Depression and low self-esteem are influenceable
	**K1.6b** Effect of negative cognitive styles on mood and self-esteem			
**PO 1.7.**	**K1.7** Importance of positive activities		**S1.7** Pursue positive activities [Behavioral activation/Commitment]	**B1.7** Positive activation is indispensable for my mental health
**Behavioral outcome 2: reduces reactivity to internal experiences [Cognitive defusion]**				
**PO 2.1.**	**K2.1** Effects of maladaptive coping strategies (submission, control or avoidance) on thoughts		**S2.1** Anticipate consequences [Expectancy reasoning]	**B2.1** The problem is not the symptom, but how I react to it
**PO 2.2.**	**K2.2a** Biographical influences on thinking patterns		**S2.2** To understand connections and concepts of psychological constructs [Information processing]	**B2.2** Thoughts, delusions and hallucinations are merely words and pictures inside my head
	**K2.2b** Conceptualization of hallucinations as externalized loud thoughts			
**PO 2.3.**	**K2.3a** Rational behind mindfulness	**A2.3** Internal and external stimuli in the present moment	**S2.3** Allow distressing internal experiences to come and go [Mindfulness/Acceptance]	**B2.3a** I can accept the presence of difficult internal experiences
	**K2.3b** Steps to mindfulness			**B2.3b** Internal experiences come and go
**PO 2.4.**	**K2.4a** Features and effect of helpful vs unhelpful internal experiences	**A2.4a** Internal experiences	**S2.4** Select helpful internal experiences against the background of own goals [Goal-orientated action planning]	**B2.4a** The mind is not always my friend
		**A2.4b** Goals and values [Goal setting]		**B2.4b** I have the choice between reacting and not reacting to internal experiences
**PO 2.5.**	**K2.5a** Difference between fusion and defusion	**A2.5a** Internal experiences	**S2.5** Decenter from internal experiences [Self-regulation/Deliteralization/Disidentification]	**B2.5** Internal experiences don’t have the power to control my life
	**K2.5b** Defusion strategies	**A2.5b** Maladaptive coping strategies (Experiential avoidance/Thought suppression/Self-focused attention)		
	**K2.5c** Steps of defusion			
**Environmental outcome 3: psychological therapy (PT) supports recovery of individual [Social support]**				
**PO 3.1.**	**K3.1a** Importance of PT in the treatment of mental health problems	**I3.1** Socially supported by psychotherapeutic relationship [Therapeutic alliance]	**S3.1** Engage in therapy [Motivation]	**B3.1** PT is important for my recovery process
	**K3.1b** Possibilities to access PT			
**PO 3.2.**	**K3.2** Simple disturbance models and coping strategies		**S3.2a** Follow cognitively in psychotherapy sessions [Perceived competence]	**B3.2** PT is comprehensible, helpful and even fun
			**S3.2b** Overcome difficulties encountered in therapy [Self-efficacy]	
**PO 3.3.**	**K3.3** Possibilities to seek social support	**I3.3** Comfortable within the group [Group conformity, Group identity, Group norms]	**S3.3** Interact positively with fellow patients [Sense of belonging/ Collaborative problem solving]	**B3.3** I am not alone with problems
**PO 3.4.**	**K3.4** Recovery based model of illness	**I3.4** Positive about self [Self-acceptance]	**S3.4** Speak confidently about own illness [Self-confidence/Self-esteem]	**B3.4** Having mental problems doesn’t mean I am worthless
**PO 3.5.**	**K3.5a** Personal set of coping strategies to manage everyday life challenges	**I3.5** Inspired by therapist model and fellow patients [Modeling]	**S3.5** Practice new behavior outside of therapy session [Motivation/ Perceived competence/ Self-management]	**B3.5** Behavior change is possible
	**K3.5b** Importance of practicing new behaviors			

PO, performance objectives (see Table 1). Change objectives are coded according to change dimensions: Knowledge (K), Awareness (A), Skills (S), Beliefs (B), Social influences (I). If suitable, change objectives were labeled with the appropriate change mechanism that can be found in the square brackets.

### 2.4. Step 3: evidence-based change methods

In Step 3 of IM, we used our matrix of change to link our change objectives to so called change methods. Change methods describe theory-based behavior change techniques (BCTs) (69) that are believed to influence change objectives (e.g., knowledge increase may be achieved through the change method psychoeducation) (69). Instead of asking *Why does change occur?* we were now concerned with the question *How does change occur?* (43). We selected our evidence-based change methods from various literature resources (70, 71) including IM’s comprehensive taxonomy of BCTs (43, 69) and translated them into practical applications. A practical application refers to a therapeutic strategy derived from the change technique that can be implemented in a real-world setting (40). For example, to achieve our change objective of increasing knowledge about the impact of internal experiences on behavior, the intervention utilizes psychoeducation as a change technique. This is practically done by providing an everyday example (such as “Imagine your best friend doesn’t call on your birthday”) (72) (p. 104) to the patients and asking them how they might feel, think, and react (73). Practical applications were informed by existing mechanism-based intervention practices for (acute) settings as identified in Step 1 (8, 18, 20, 22, 23, 59). The final output for Step 3 comprised of a matrix of change methods containing all procedures planned to be incorporated into our intervention (43) (see Table 3).

**TABLE 3:** Matrix with change methods/techniques and practical applications (Step 3).

Change objectives	Behavioral change techniques	Practical applications
**Increase knowledge**	Conscious raising; Persuasive communication; Discussion; Elaborating; Scenario-based risk information; Psychoeducation	Therapist-led information input (verbal; written; visual) e.g., on cognitive biases; group brainstorming and discussions
**Raise awareness**	Self-monitoring; Thought-monitoring; Introspective training; Using imagery/analogy; Behavioral experiments; Directing attention; Mindfulness training	Therapist-asked prompted questions (e.g., “Image a friend doesn’t call on your birthday; how would you feel?”); thought records; guided mindfulness exercises e.g., Leaves-on-a-river mediation; using metaphors to explain selective attention e.g., attention like a spotlight just focused on one information
**Change beliefs**	Belief selection; Persuasive communication; Active learning; Cognitive restructuring	Therapist-led summary at the end of each session (e.g., learning objective: “Always think through several possibilities that could contribute to a situation or event!”); Take-home rounds (“What was important for you today?”)
**Improve skills**		
– S1.1 Report internal experiences	Introspective training	Therapist-asked explorative questions (e.g., “What came into your mind when you saw this picture? How would you feel if your opposite doesn’t greet you?”); Entrance rounds (“On a scale of 1 to 10; how are you feeling today?”); mindfulness exercises
– S1.1/S2.1 Anticipate consequences	Conscious raising; Self-reevaluation	Therapist-led information input (verbal; written; visual); group brainstorming and discussions; therapy cards with prompting questions (e.g., “Even if I am right; Am I overreacting?”)
– S1.3 Rationally analyze	Arguments; Shifting perspective; Direct experience; Reattribution training; Cognitive restructuring; Critical reasoning	Therapist-led group exercises to contemplate on different causes of events (e.g., “People are laughing while you are talking. What might be the reason?”); sharing of personal examples in group
– S1.4a Gather information – S1.4b Challenge conclusions	Arguments; Shifting perspective; Direct experience; Decision making; Critical reasoning	Therapist-led group exercises to gather enough information before drawing conclusions (e.g., “A fellow patient doesn‘t acknowledge you when you walk past each other. Did she ignore you on purpose?”); sharing of personal examples in group
– S1.5a Consider context – S1.5b Take perspectives – S1.5c Tolerate ambiguity	Environmental reevaluation; Arguments; Shifting perspective; Direct experience; Empathy training; Critical reasoning; Social cognitive training	Therapist-led group discussion on social cues for social reasoning; group exercises to gather enough information before drawing conclusions (e.g., “During an appointment; the doctor has a serious expression and an intense stare. Why?”); sharing of personal examples in group
– S1.6 Come up with functional thoughts	Deconditioning; Reframing	Therapist-led group exercises to come up with more helpful thoughts for different events (e.g., “You fail an exam and your mind immediately tells you that you are a failure. What would be a more helpful appraisal?”); sharing of personal examples in group
– S1.7 Pursue positive activities	Behavioral planning; Activity scheduling	Therapist-led group brainstorming on positive activities; participants choose one activity and schedule it for the upcoming week
– S2.2 Understand psychological constructs	Elaboration	Therapist-led information input on psychological formulation of psychotic symptoms and group discussion
– S2.3 Allow distressing internal experiences	Acceptance training; Mindfulness training	Therapist-led behavioral experiments to demonstrate counterproductive effect of thought avoidance e.g., Don’t-think-of-the-pink-elephant; mindfulness training e.g., mindfully-eating-a-raisin
– S2.4 Select internal experiences	Using imagery; Self-affirmation; Goal setting; Disputation	Therapist-led practical exercises and metaphors e.g., Bad-cup/Taking-your-mind-for-a-walk; functional disputation e.g., “Is this thought helpful?” and goal clarification (e.g., “What is important for you in this situation?”)
– S2.5 Decenter from internal experiences	Active learning; Using imagery; Counterconditioning; Planning coping resources; Training executive functions; Guided practice; Self-monitoring; Attentional training; Self-Instruction Training	Therapist-led practical defusion exercises e.g., Labeling-your-thoughts; group discussion and selection of individual techniques
– S3.1 Engage in therapy	Motivational interviewing; Participating problem solving	Therapist directly approaches new patients; explains advantages/disadvantages of PT; develops joint therapy goals
– S3.2a Follow cognitively – S3.2b Overcome difficulties	Cognitive training	Therapist ensures that contents are in a simple and comprehensive form; adapts each session according to cognitive level; challenges participants with exercises; includes fun activities
– S3.3 Interact with fellows	Interpersonal contact	Therapist ensures secure group framework (group rules and mediation in the case of problems); Therapist-led group discussions and reflections; encouragement of personal group exchange
– S3.4 Speak about own illness	Interpersonal contact; Shifting perspectives; Reframing; Cooperative learning	Therapist holds and attitude of destigmatization; normalizes psychotic experiences; encourages sharing of personal experiences
– S3.5 Practice behavior	Behavioral rehearsal; Set homework tasks; Self-help	Therapist suggests homework assignments and gives space for debriefing
**Encourage positive social influences**		
– I3.1 Socially supported	Mobilizing social support/networks; Social support theory; Increasing stakeholder influence; Social skills training	Therapist shows empathy and understanding, regardless of dysfunctional behavior; repeatedly offers relationship despite initial rejection
– I3.3 Comfortable in group	Interpersonal contact; Participatory problem solving; Entertainment education; Forming coalitions,	Therapist ensures secure group framework; reinforces participation and group exchange
– I3.4 Positive about self	Verbal persuasion; Stereotype-inconsistent information; Reducing inequalities of class/race/gender and sexuality; Provide contingent rewards	Therapist praises participation; is open to different points of view and does not judge participant’s internal experiences
– I3.5 Inspired by therapist and fellows	Modeling; Cooperative learning	Therapist encourages sharing of personal experiences; gets involved with personal examples e.g., “I know that feeling. My mind always tells me that I am not good enough.”

Each change objectives can be found in the matrix of change (Table 2). Change objectives from the domains knowledge, awareness and beliefs were each combined into one major change objective due to overlap. Change objectives found in the dimension skills and social influences on the other hand were all treated separately. Behavioral change techniques are taken from IM’s comprehensive taxonomy of BCTs (43, 69).

### 2.5. Step 4: intervention outline

In line with our intervention draft of Step 3, we designed treatment modules, the associated sessions (see Table 4), produced therapy materials and decided on our delivery format outlining therapy frequency and duration of sessions. Next to creating completely new materials, we made sure to thoroughly examine existing therapy manuals for usable parts. If some materials of an intervention were suitable, we made adjustments before integrating them into our intervention. During the development process, project team members and independent clinical fellows constantly reviewed materials and session outlines. We also made sure to carry out some informal test-runs with patients whose verbal feedback was used to revise session contents for the final intervention that is currently tested in a feasibility study.

**TABLE 4:** Table giving an overview of the objective and core exercises for each session of MEBASp (Step 4).

Session	Title, main objective and target change mechanism	Core exercises and metaphors
1.	**Psychoeducation** Objective: Understanding the cognitive model, awareness of problematic cognitive biases and over identification/reaction to them Target mechanism: Knowledge increase	Developing theory based on an everyday example (“Imagine your friend doesn’t call on your birthday”) and interactive group discussion Source: MCT for depression (72)
**Module cognitive insight [Metacognitive knowledge and awareness]**		
2.	**Finding explanations** Objective: Changing dysfunctional attributional patterns by understanding that multiple factors can lead to a scenario Target mechanism: Attributional reasoning	Contemplating different causes for everyday examples and discussing negative consequences of monocausal attributions Source: MCT for psychosis and MCT-acute (21, 121)
3.	**Jumping to conclusions** Objective: Avoiding premature first impressions, adjusting conclusion when new information emerges Target mechanism: Interpretative reasoning	Holding back and revising premature decisions with the help of various fragmented picture tasks where patients have to guess the object behind it Source: MCT for psychosis and MCT-acute (21, 121)
4.	**To empathize** Objective: Understanding that facial expressions can easily be misinterpreted, considering various information sources when assessing your opposite Target mechanism: Social reasoning	Trying to guess what a person may feel or intends to do by judging pictures of their faces and discussing everyday examples Source: MCT for psychosis and MCT-acute (21, 121)
5.	**Mood and self-esteem** Objective: Recognizing dysfunctional thinking styles, finding alternative views and engaging in positive actions Target mechanism: Cognitive reappraisal	Gathering symptoms of depression, finding more helpful thoughts for negative cognitive schemas in various everyday examples, collecting positive activities to counteract depressive mood and low self-esteem Source: MCT for psychosis and MCT-acute (21, 121)
**Module cognitive defusion [Metacognitive goals and strategies]**		
6.	**Noticing thoughts** Objective: Being more present in the moment, noticing inner and outer sensations and responding more consciously to them Target mechanism: Mindfulness	Practicing mindfulness for external (mindfully eating chocolate) and internal (observing thoughts) experiences, metaphors: “life on autopilot,” being a “distant observer” Source: ACT for psychosis (158)
7.	**How our mind works** Objective: Developing a different relationship toward thoughts by understanding that they mostly consist of automatic rules and judgments learned in our past, giving thoughts less power dictating our behavior Target mechanism: Goal-orientated action planning	Debunking thoughts by distinguishing between facts and appraisals (Bad Cup), noticing automaticity and uncontrollability of thoughts (“Mary had a little lamb” and “Don’t think of a pink elephant”) and acting contrary to thoughts (“Don’t do what your mind says”), metaphors: mind as a “production machinery” and “hard drive” with “data garbage” Source: ACT metaphors (159) and ACT for life (160)
8.	**Helpful vs. unhelpful thoughts** Objective: Distinguishing between helpful and unhelpful internal experiences and learning to act contrary to them without trying to avoid or control them Target mechanism: Disidentification	Classifying everyday thoughts in unhelpful and helpful thoughts, actively executing defusion in “Taking your mind for a walk,” metaphors: thoughts as “ankle cuffs” vs. “tools” Source: ACT for psychosis (158)
9.	**Defusion techniques** Objective: Learning to actively distance from internal experiences by using cognitive and behavioral strategies Target mechanism: Self-regulation	Trying out different defusion and detached mindfulness techniques e.g., “labeling thoughts,” “floating leaves on a stream” and “Attention training technique” and choosing one for the “instruction manual for the mind,” metaphors: mind as “parrot” always telling the same story, the little “mind monster” Source: ACT metaphors (159), ACT for psychosis (158), Metacognitive Therapy for anxiety and depression (122)

### 2.6. Step 5 and 6: implementation and evaluation plan

After completing step 1–4, IM includes two additional steps consisting of setting up an implementation and evaluation plan (43). However, we decided to follow van Agteren and colleague’s decision to exclude these steps in our current research (40) as this allowed us to provide a more detailed insight into our intervention development process. Nevertheless, the evaluation of the intervention is covered by the above mentioned feasibility study (clinicalTrials.gov identifier: NCT04874974) (74). We will give a brief overview of our ongoing pilot study in the future directions part of the discussion section.

## 3. Results

### 3.1. Step 1: logical model of the problem and needs analysis

To facilitate a deeper understanding of our initial project phase and literature research, we present the results of Step 1 in a narrative format that begins with a brief description of our development context and population and progresses to the problem definition and the derivation of needs.

#### 3.1.1. Development context and target population

MEBASp is part of a research initiative at the Max Planck Institute of Psychiatry in Munich, Germany, which aims to implement a clinic-wide mechanism-based treatment concept containing different group modules each focusing on a specific change mechanisms like emotion regulation or behavioral activation. By identifying individually relevant psychological processes and personal preferences of each patient on admission, the clinical team ensures a targeted treatment selection by combining indicated therapy modules (9, 75–77). In this context, our IM approach focused on the development of an intervention targeting change mechanisms found to be relevant in acute inpatients with positive and/or negative psychotic symptoms (according to ICD-10 criteria) treated in an (locked) acute psychiatric ward (78) (for a detailed research background on the concept see Supplementary Methods and Supplementary Figure 1). Based on the assumption of psychosis as an independent clinical trait (79), our target inpatient group covered the entire psychosis-spectrum as well as psychotic depression and psychotic bipolar disorder.

#### 3.1.2. Defining the problem of acute inpatients with psychotic symptoms

In the course of our epidemiological assessment, we specified two main mental health problems for acute inpatients with psychosis (16, 25): (1) severe positive symptoms such as hallucinations and delusions and (2) resulting dangerous behaviors toward themselves and others making immediate (compulsory) hospitalization necessary. Our social assessment in turn revealed a tremendous negative impact of the severity of positive symptoms and crisis-associated hospitalization on patients’ QoL (27, 80–82). Both are believed to contribute to the secondary activation of negative symptoms such as poor rapport (83) and comorbid disorders like mood and anxiety disorders (84) resulting in an increased chronification risk (85). Relevant contributing psychological processes in the development of negative symptoms thereby seem to be a demoralization due to patients’ low expectancies for pleasure or success (64, 86), internalized stigma (87), a lack of participation and activities (88), and maladaptive coping responses such as social anhedonia and substance abuse to deal with aversive internal and external experiences (89).

Furthermore, our ecological assessment (see Supplementary Table 2) identified metacognitive deficits (90) to be the main determinant for pathogenetic cognitive processes associated with positive symptoms (first health problem). We also found associations of metacognitive deficits with negative symptoms and impaired processes discussed above (91). While metacognition is being broadly defined as “knowledge about knowledge” (92), it can be further distinguished into a knowledge (knowledge and beliefs about cognition), an awareness (conscious experience of and reflection about cognitive processes), a goal (setting goals on a meta-level), and a strategy part (conscious application of functional strategies for goal achievement) (93). Patients with psychotic symptoms thereby seem to show deficits in all four components (94). Deficits in metacognitive knowledge and awareness moreover are believed to lead to cognitive distortions (e.g., jumping to conclusions, attributional biases, theory of mind deficits) (60), dysfunctional beliefs and expectancies (associated with a low self-esteem and negative symptoms) (10, 95) and a lack of cognitive insight into those cognitive biases (96). For instance, a lack of knowledge about common human cognitive biases, poor awareness of one’s own thoughts, and the inability to recognize distortions in conclusions could lead to misinterpreting a crackling sound on a phone line as proof of being watched (21). Delusional thoughts and hallucinations alone however, do not automatically result in distress and dysfunctional behavior making compulsory hospitalization necessary (second health problem). It seems to be the appraisal and behavioral reactivity toward the thought and voice contents that increases the probability of danger to self and others (97). Psychological processes linked with this problem are cognitive fusion with internal experiences and maladaptive coping strategies such as experiential avoidance, thought suppression and worry (66, 98–100). Explained in highly simplified terms, cognitive fusion describes a cognitive process in which a person is fully entangled with the verbal content of internal experiences, beliefs it to be true and reacts to the content (101). Consistent with metacognitive process models, cognitive fusion can be associated with a deficit in metacognitive goal setting and strategies leading to the increased reactivity to dysfunctional thought content (102). The idea of being surveilled may e.g., take on great importance due to dysfunctional metacognitive beliefs, such as that one’s thoughts are true and need to be acted on. Without being aware of own goals and values, one may turn to dysfunctional coping strategies like aggression, social withdrawal or excessive worrying, which in turn can escalate into mental crisis followed by a decrease in functioning (103). In summary, there is convincing evidence for the contribution of metacognitive deficits to both severe psychotic symptoms and subsequent crisis development (104).

Lastly, our policy assessment identified bio-social vulnerabilities and structural (health) system barriers that lead to environmental risk factors such as social conflicts (interpersonal), a general shortfall of psychosocial treatments (organizational), stigma and societal disadvantages (society) that all seem to additionally contribute to our overall problem (105–107). For a visualization of our problem theory see Figure 2.

#### 3.1.3. Determining the needs for development and implementation

Having a better understanding of our problem and the underlying impaired processes, we were now able to draw general implications for the implementation of the intervention itself.

Considering the severity of mental health problems and low QoL, we first of all determined a great need to generally expand and improve the psychotherapeutic offer for acute inpatients with psychosis. Although guidelines recommend psychological care already in the acute illness phase (3, 4), implementation rates on acute ward are still extremely low (28, 108, 109) resulting in a dissatisfaction among patients who criticize the predominant pharmacological and risk-focused treatment (27, 110). The demand for psychosocial treatments that do not involve medication but aim to assist with recovery, on the other hand, is high (25).

The second need we derived was the necessity to adapt existing mechanism-based interventions to the specific characteristics of acute ward and inpatients with psychotic symptoms (25). Available concepts are often lengthy and quite complex in content and it has to be doubted if they can actually work efficiently in acute settings (6, 16, 28). Main limitations consist of short hospital stays (111) and patients’ general difficulties to engage with traditional psychotherapy concepts due to treatment resistance (112, 113), high distrust levels toward the entire environment (114), emotional distress (115), severe cognitive deficits (116), and dual diagnoses (117). Despite the demanding patient clientele, therapists in acute settings are moreover challenged to provide psychological therapies with minimal resources (16). Staff shortage, economic pressure and administrative duties leave little room to offer individual therapy to each patient making group-based formats a cost-effective alternative to reach a large number of patients (118). Moreover, group interventions offer valuable opportunities for interpersonal skill development and peer support (26). Due to high patient turnovers, group therapies should be delivered in standalone formats with patients being able to already benefit when attending only one session or one module (118). Despite the economic and social benefits of group concepts, it is advisable to offer at least a minimum number of individual sessions to provide additional space for addressing personal needs and topics (119).

Thirdly, we formulated the need to consider both staff and patients’ needs when planning the content of the intervention. While care taker priorities often focus on symptom and risk management, patients themselves name social circumstances and intra- and interpersonal symptom distress (e.g., unwanted internal states, sleep difficulties, lost sense of identity, social isolation, and stigma) as their main concerns (16, 25, 28, 120).

In summary, our findings suggest that an effective and feasible intervention for acute psychiatric inpatients should focus on key mechanisms associated with changes in symptom severity and patients’ symptom distress. A group concept is favored over individual sessions due to economic and social reasons, although additional individual sessions should be offered based on individual needs or demand. Therapy sessions should be simple, brief, flexible, low key, and able to be delivered stand-alone.

#### 3.1.4. Examining existing practice

Beside Metacognitive Training (MCT), we identified two more mechanism-based therapies for psychosis focusing explicitly on impaired metacognitive processes linked to our first health problem (positive symptoms) (36): Metacognitive insight and reflection therapy (MERIT) and Metacognitive interpersonal therapy for psychosis (MIT-P). However, sufficient evidence was only available for Metacognitive Training (37–40) that furthermore recently provided an open-source transdiagnostic group format suitable for acute inpatients and acute settings (121). The concept of MCT by Moritz and Woodward was originally inspired by research on cognitive biases in psychosis (65) and aims to convey metacognitive knowledge and raise metacognitive awareness for dysfunctional thought patterns (60, 122). Compared to Metacognitive Therapy by Wells and Matthews, MCT not only focuses on general thinking mechanisms from a metacognitive perspective, but also on specific thoughts from a cognitive one by directly addressing thought contents (60). MCT’s goals are implemented in a group therapy format that works with non-confrontational, educative and delusional-neutral material (21). Although MCT was originally developed for psychosis, it has been adapted for use in treating other disorders such as depression and personality disorders and can be applied in a transdiagnostic manner (60).

Our target processes cognitive fusion and maladaptive coping strategies related to our second health problem (dangerous behaviors and hospitalization) on the other hand are the main subject in the Acceptance and Commitment Therapy by Hayes (101) and the Metacognitive Therapy by Wells and Matthews (122). In contrast to traditional CBT principles of disputation and restructuring, ACT focuses on transdiagnostic change mechanisms such as acceptance and cognitive defusion to modify patients’ relationship toward internal experiences changing their function on behavior (19). Defusion thereby refers to a decentering-related mechanism that operates through metacognitive goal clarification (e.g., asking yourself if this thought is helpful for your broader goals and values) and the use of mindfulness-based distancing strategies (123). Similar to defusion techniques, Well’s Metacognitive Therapy aims to reduce toxic thinking styles such as worry and threat monitoring believed to maintain paranoid thoughts and hallucinations by changing dysfunctional metacognitive beliefs and practicing metacognitive strategies like detached mindfulness (124). Both ACT and Metacognitive Therapy share their transdiagnostic orientation and focus on metacognitive strategies and have demonstrated effectiveness in working with psychosis in smaller studies (18, 19, 59, 103, 124, 125). However, most studies were either conceptualized for individual therapy and/or outpatients (5–7) with most available concepts still rather unsuitable and demanding for group inpatient settings. For an overview and further description of differences between treatments and key change mechanisms see Supplementary Table 3.

### 3.2. Step 2: intervention outcomes, change mechanisms, and logical model of change

Looking at each target area of our problem model, we formulated desired intervention outcomes and constructed a logical model of change (see Figure 3) linking outcomes and hypothesized mechanisms of change. As we were challenged to address the very diverse needs of our target population in one intervention, we made sure to come up with treatment goals applicable to a wide range of mental health problems. Following the ACT nomenclature, we therefore no longer speak of specific symptoms such as delusional thoughts or hallucinations, but group them together under the term distressing internal experiences (97).

Our overarching treatment goal was to encourage functional behavior and coping via improving *cognitive flexibility*. Cognitive flexibility thereby can be understood as the capacity to switch between cognitive processes in order to generate effective behavioral regulation and is determined by general metacognitive abilities (126). To achieve this objective, we aimed to raise patients’ *cognitive insight* on cognitive distortions and hence the patients’ capability to reflect on internal experiences on a meta-level (127, 128). Cognitive insight is linked to functional *metacognitive knowledge and awareness* and has been identified as a potential promising candidate mechanism for a decline of positive symptoms in psychosis and favorable treatment outcome in other disorders (127).

Furthermore, we aimed to reduce patients’ reactivity to aversive internal experiences via promoting *cognitive defusion*, which is determined by functional *metacognitive goals and strategies*. Cognitive defusion has been found to generally improve functioning, reduce dysfunctional attitudes, anxiety, negative affect (102) and also post-traumatic-like symptoms (129) and sleep difficulties (130). It has also been found to mediate symptom distress in psychosis via reduced believability of thought and voice content (131), and changes in *metacognitive beliefs* and *coping skills* (18).

Lastly, our intervention was supposed to support patients’ recovery by providing positive *social support* and with this foster peer group relationships, and a strong therapeutic alliance found to be essential ingredients for treatment success (132, 133). Overall, we hoped that our identified transdiagnostic change mechanisms and outcomes would support patients in a wide range of needs and topics, thus improving their mental health and QoL in the long term and prevent or at least mitigate further relapses.

We then divided all intervention outcomes into performance objectives (PO) (see Table 1), which we subsequently linked to our overarching change dimension via change objectives in our change matrix (see Table 2).

### 3.3. Step 3: evidence-based change methods

All change objectives were assigned to change techniques and practical applications in our matrix of change methods (see Table 3). The main change methods in our intervention blueprint consisted of therapeutic techniques fostering knowledge increase, introspection, perspective-taking and cognitive/behavioral regulation (69). As we faced the challenge to translate a complex set of change objectives and methods into very simple and comprehensible end applications for a group format, we made sure to come up with lots of interactive information sharing and fun exercises inspired by techniques used in existing mechanism-based interventions such as MCT, ACT and Metacognitive Therapy (see Step 1). For the change objective “Patient is able to allow distressing internal experiences” we for example planned to integrate a mindfulness training by performing simple guided exercises such as the “Leaves-on-a-river” from the ACT for psychosis manual (134).

### 3.4. Step 4: intervention outline

#### 3.4.1. Transdiagnostic conceptualization

Although our intervention development aims to target mainly change mechanisms behind psychotic symptoms and crisis development trough symptom distress, the identified underlying impaired processes are interrelated with several other disorders (see Supplementary Table 2). Metacognitive deficits (135), cognitive distortions (61), a lack of cognitive insight (136), and cognitive fusion (137) for example play an important explanatory role among others in anxiety, mood, personality disorders, and substance abuse (138). Cognitive insight, cognitive defusion, social support, cognitive flexibility, and in turn improved metacognitive skills are considered to function as transdiagnostic mechanisms of change in therapy (123, 139–141). Hence, our transdiagnostic concept allows us to address not only the different needs of our patients with psychotic and comorbid diagnosis, but also patients with diagnoses other than psychosis. Given the heterogeneous patient composition of acute ward, a transdiagnostic mindset and approach might be an especially valuable treatment component (24).

#### 3.4.2. Modules and sessions

Our final intervention comprised a 5-week group therapy program consisting of three short treatment modules and a total of nine sessions.

Module I (Psychoeducation) gives a brief introduction into the rational of the therapy and the targeted change mechanisms. The terms cognitive distortions and cognitive fusion and their role in the development of general psychological problems are explained in a simple language and with the help of examples and small exercises. The importance of cognitive insight and cognitive defusion for mental health is made clear.

Module II (Cognitive Insight) consists of four sessions and aims to raise cognitive insight by explaining and illustrating different cognitive biases and demonstrating their negative consequences on mental health. The treatment module includes materials and interventions adapted from the MCT for psychosis, MCT for depression and MCT for acute psychiatric settings (MCT-acute) and focuses on the change domains metacognitive knowledge and awareness.

Module III (Cognitive Defusion) with another four sessions aims to change the function internal experiences have on the patient’s behavior by strengthening adaptive coping strategies. Exercises are assembled from various ACT and Metacognitive therapy manuals and cover the change domains metacognitive goals and strategies. An overview of the intervention’s contents and sources for used materials can be found in Table 4.

All sessions follow the same general procedure: entrance round with mood poll, brief introduction to the program and group rules, experience-based exercises and group discussions, linking therapy content to mental health problems, transferring knowledge into every-day life, take-home message and closing round.

#### 3.4.3. Delivery format and framework

**FIGURE 4:**
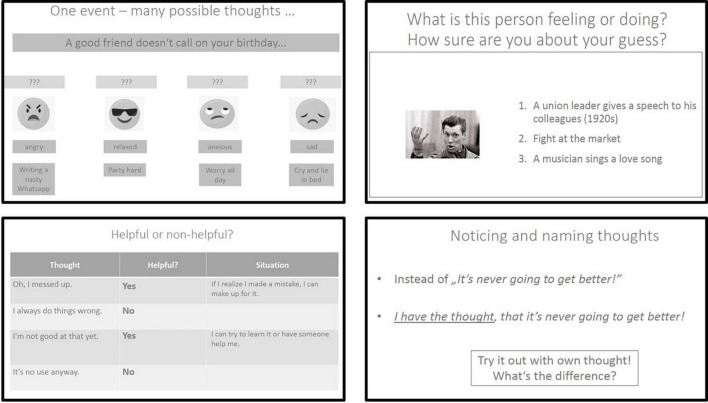
Example slides from each module. **Top left**: Slide from the module “Psychoeducation”. Patients learn to understand that different thoughts can lead to different feelings and behaviors (slight adapted from the MCT manual for depression) (72) (p. 105). **Top right**: Slide from the module “Cognitive Insight” and session “To empathize”. Patients learn to understand that facial expressions can easily be misinterpreted (slide used from the MCT-acute concept, open source on the MCT website, https://clinical-neuropsychology.de/metacognitive_training/). **Bottom left**: Slide from the module “Cognitive Defusion” and session “Helpful vs. unhelpful thoughts”. Patients learn to distinguish between helpful and unhelpful internal experiences. **Bottom right**: Slide from the module “Cognitive Defusion” and session “Defusion techniques”. Patients learn to notice and name thoughts in order to create distance to them instead of getting entangled in thought contents and automatic reactions.

We propose group therapy takes place twice a week with each session lasting between 40 and 60 minutes depending on the group’s cognitive capacity. To ensure a maximum of flexibility for patients with brief treatment duration and attendance preferences, all modules are independent from each other and each session can be delivered stand-alone. Information is presented on simple PowerPoint slides with plain language, short inputs and illustrating imagery makes participation possible even for patients with pronounced cognitive impairments. Simple metaphors, concrete and personally relevant experience-based exercises and “touchable” therapy material (e.g., bringing dark sunglasses to demonstrate the information filter of our mind) make contents additionally easy to understand and create a relaxed atmosphere (97). All patients receive a patient workbook with short session summaries, exercises and optional homework assignments. Two therapy-cards in pocket size summarize the most important points of each module. See Figure 4 for therapy content examples. Due to high levels of distress and occasionally hostile and suspicious behaviors, group sizes are kept small with a maximum of seven participants. Group sessions can be carried out by a clinical psychologist, psychiatrist, trained nurse or an occupational therapist, as little prior knowledge is required because of its simple conceptualization and available therapy manual. Next to group therapy, we advise all patients receive psychosocial treatment-as-usual (see Supplementary Methods) and additional individual psychotherapy sessions.

#### 3.4.4. Therapeutic attitude

The therapists general therapeutic attitude should be empowering trying to support patients to pursue their valued goals despite symptoms of serious mental illness (28, 97). They should moreover try to create an open, acceptance-based and destigmatizing atmosphere (142). The therapists’ process-oriented stance, which sees psychotic symptoms as extreme manifestations of normal human cognitive distortions and dysfunctional strategies, can thereby foster rapid alliance building (21). Self-disclosure by therapists is strongly recommended at this point, as it allows them to convey to patients that they too are often “victims” of their own cognitive biases (97). Thereby, they work in accordance to key features of third-wave therapies that place therapists on an equal level to patients in the sense of “you cannot teach what you cannot do” (9) (p. 369). A focus on mechanisms of change rather than symptom disputation moreover reveals room for change and returns a sense of control to patients (10).

Group attendance is voluntary, however, participants should be personally approached before each session to encourage participation (28). During sessions, patients have the possibility to leave the group if they feel uncomfortable as well as the option to return. Contents of psychotic thoughts and experiences can be talked about openly without being judged as wrong, right or even pathological (142). Therapy sessions should not be rushed and therapists should give enough time for discussion and exchange between the participants. They can promote involvement by directly approaching patients with simple questions and thus encouraging socially anxious participants. Following the transdiagnostic concept of the intervention, disease-related language is rarely used (143).

## 4. Discussion

The current research aimed to develop a novel mechanism-based therapy for acute inpatients with psychotic symptoms using Intervention Mapping as a structured development framework to improve the intervention’s scientific foundation, reporting standards and potential reproducibility. To our knowledge, this is the first research for this specific setting and patient group, which has attempted to do so.

MEBASp is a low-threshold transdiagnostic and modularized group therapy that focuses on symptom and distress reduction and responds to a central priority of health care services to develop, test and offer effective and needs-oriented care for acute inpatients with psychosis (5–7). We believe that our underlying intervention model and format will be able to meet the complex needs of those patients and the settings they are treated in due to several reasons.

Firstly, our intervention directly targets hypothesized change mechanisms instead of specific symptom content and hence follows a current promising paradigm shift in intervention science toward process-based treatments (13, 75, 144). We believe that our mechanism focus will not only allow us to optimize patients’ treatment outcomes (13), but will be especially helpful when working with acute (involuntary) inpatients. As suggested by Moritz and Woodward (21), MEBASp operates through a non-confrontational and symptom-neutral “backdoor” approach (p. 623) that could be beneficial to address a transdiagnostic spectrum of patients and diverse needs, foster rapid alliance building, motivate resistant patients, lower drop-out rates, and enhance recovery rather than illness elimination (6, 16, 19, 145). By combining evidence-based mechanisms and procedures from various theories and therapy schools into one approach, we moreover refocus on key questions of why and how interventions work best for patients instead of if they align or differ from specific therapy approaches (75).

Our intervention’s overarching emphasis on transdiagnostic metacognitive change mechanisms (cognitive insight and cognitive defusion) furthermore fits in a new generation of treatments promoting recovery from serious mental illnesses including psychosis (104, 138). The concept of metacognition thereby is believed to serve as a valid candidate for filling the gap between simplistic biological treatment models and psychosocial ones (104). A main benefit of metacognitive treatment models is the promotion of overall wellbeing beyond the positive symptom reduction achieved through psychopharmaceuticals, an aspect considered to be essential when working in psychiatric inpatient care (27). However, authors criticize that existing treatments only cover certain aspects of the larger construct of metacognition (see Supplementary Table 3) (93) and call for intervention development that incorporate all four metacognitive domains into hybrid approaches (104). Due to our modularized treatment concept, MEBASp is actually able to enclose the whole spectrum of metacognitive mechanisms into one intervention. Patients therefore not only benefit from a broad range of hypothesized positive treatment effects when attending all three modules, but already profit when attending only one or two (76).

Despite an underlying change theory seeming complex at first sight, we moreover believe that we managed to adapt the intervention for the inpatient context. MEBASp is brief, flexible, experience oriented, low-key and easy to learn for therapists and thus takes into account key treatment elements proposed by competence frameworks in working with acute patients (26, 58). The modularized approach moreover allows to combine and integrate different independent treatment modules and therewith ensures high flexibility and goodness-of-fit to patient needs and preferences (146). All procedures taken from in- and outpatient concepts are simplified and adapted for a crisis-focused setting addressing both priorities of symptom (cognitive insight) and distress reduction (cognitive defusion) (25). On top of that, the group-based design permits high therapy frequency and dose, is cost-effective, resource saving and offers opportunities for peer social support and interpersonal skill development (147).

### 4.1. Advantages to the IM approach

Although the research base on process-oriented care is growing, authors do not yet provide a standardized method on how to construct evidence-based problem models, choose adequate sets of change mechanisms, procedures and change measures (13, 148). In this context, IM offers different structured elements to overcome those challenges. The PRECEDE-model allowed us the synthetization of multi-level data and an in-depth understanding of our situation necessary for identifying evidence-based change mechanisms (31). Building matrices of change and change procedures represented a valuable method to ensure our change mechanisms were precisely defined (148) and got effectively linked to therapeutic applications (75). In doing so, we could refer back to IM-associated extensive frameworks such as the Theoretical Domains Framework (68) and the taxonomy of behavior change methods (69) that clearly close the gap of comparable compositions in the literature (144). Thereby, IM per se works according to principles of mechanism-based therapies by being “theory agnostic,” flexibly combining evidence-based concepts from across paradigms and thus creating synergistic effects between different approaches (149). Lastly, the detailed mapping of all change mechanisms and procedures in an intervention blueprint reflects the underlying complexity of our intervention and allows the derivation of matching outcome measures to monitor change in future studies (as described in the future direction sections) (148).

### 4.2. Limitations

There are several limitations to the current research. First, the mechanism and procedure selection were based on considerations and decisions made by our development team in each step of the IM framework. A different working group could have created a different needs analysis and chosen a different treatment focus, change methods and practical applications (see for example the CRISIS-, the WIT- or the OASIS-study) (150–152). Nevertheless, thanks to our detailed documentation of each decision step, potential differences become transparent and are made objectifiable.

Second, we encountered an excessive concept overlap in the literature (148). Cognitive defusion for example shares significant variance with constructs such as deliteralization, decentering, distancing and detached mindfulness (102). Moreover, the concept of metacognition is also somewhat “blurry” making it difficult to separate accurately what is a metacognitive and what a purely cognitive change mechanism (153). A central source integrating processes, mechanisms and procedures and using a common language and conceptualization would have made our selection much easier and the final intervention potentially more comparable with other mechanism-based treatments.

Thirdly, the complex set of mechanisms underlying the intervention could be seen as a challenge. From a clinical perspective, an intervention focusing on trying to change such a variety of mechanisms might be an overload for acute inpatients. Along with this, our mechanism-based group will naturally not provide the appropriate content and format for all patients due to varying needs and preferences. In addition to alternative therapy options (see Supplementary Figure 1), further research should investigate which patients can particularly benefit to make appropriate therapy offers.

Fourthly, due to time and resource constraints and in consideration of protecting the wellbeing our vulnerable target population, we did not conduct codesign activities during the first development stage. This decision may have limited the intervention prototype’s suitability and acceptability for patients. Although we relied on pre-existing qualitative data and plan to integrate codesign activities in the second stage of the development process (feasibility study), future research should explore appropriate and sensitive ways to involve patients already in the first development stage.

Finally, although we found the detailed approach of IM helpful in creating our intervention and followed most of its steps, the overall development process was time consuming and took up a lot of resources. If teams thus require rapid intervention development, a more pragmatic approach such as the 6SQuID (“Six steps in quality intervention development”) (154) might be favored over IM.

### 4.3. Implications and future directions

Our mechanistic treatment design enables us to conduct necessary research to determine whether our proposed mechanisms are capable of producing therapeutic change (13). A single-arm feasibility study investigating the impact of MEBASp is currently in progress (clinicalTrials.gov identifier: NCT04874974) (74). The study includes a mixed methods evaluation to assess the feasibility and test key change mechanisms of our logical model of change. Next to primary outcome measures such as trial entry rate, patient engagement and satisfaction, the study includes metacognitive measures e.g., the Beck Cognitive Insight Scale (155) and the Cognitive Fusion Questionnaire (156). Intensive involvement of participants trough codedesign activities such as feedback questionnaires, feedback rounds and interviews moreover ensures the revision of the intervention prototype will be in accordance to patients’ needs and preferences (157). If feasible and acceptable, future research will further investigate on the effects of change mechanisms by involving a control condition and performing mediation analyses in a larger scale study. Our ultimate goal is to individualize treatment allocation by matching patients to the treatment module most likely to produce change and fit with personal preferences (see Supplementary Figure 1). The allocation process could in the long term involve e.g., moderation studies, complex network approaches and ecological momentary assessments (75).

### 4.4. Conclusion

Our research demonstrates the importance of a) developing needs-oriented and mechanism-based interventions for acute inpatients with psychotic symptoms and b) using a structured development methodology to ensure their scientific foundation and replicability. Our rigorous and evidence-based intervention design focuses on addressing metacognitive change mechanisms associated with both acute symptoms and crisis development and adapts to key components required to deliver psychotherapy in psychiatric inpatient settings. It therefore has the potential to positively impact a neglected patient group. However, a pilot study is required to assess the intervention for safety, feasibility and preliminary effectiveness.

## Data availability statement

The original contributions presented in this study are included in the article/Supplementary material, further inquiries can be directed to the corresponding author.

## Author contributions

EG, SL, PF, FP, SE, and JK-B: research objectives, project methodology, needs-analysis, guidance of process and clinical input, review intervention prototype, and manuscript write-up. EG: theoretical framework for intervention, material development and beta testing with patients. All authors contributed to the article and approved the submitted version.
