# Supplementary material for: Developing a mechanism-based therapy for acute psychiatric inpatients with psychotic symptoms: an Intervention Mapping approach
Source: Front Psychiatry. 2023 Jun 1;14:1160075. doi: 10.3389/fpsyt.2023.1160075 (PMC10267344; doi:10.3389/fpsyt.2023.1160075)
Supplement: Supplementary file 1 [file Data_Sheet_1.docx]

Supplementary Material

Developing a mechanism-based therapy for acute psychiatric inpatients with psychotic symptoms: An Intervention Mapping approach

Eva Gussmann^1*^, Susanne Lucae^1,2^, Peter Falkai^1,2^, Frank Padberg^2^, Samy Egli^1^, Johannes Kopf-Beck^1,3^

^1^ Max Planck Institute of Psychiatry, Munich, Germany

^2^ Department of Psychiatry and Psychotherapy, LMU University Hospital Munich, Munich, Germany

^3^ Department of Psychology, LMU Munich, Munich, Germany

*** Correspondence:**Eva Gussmann, M.Sc.
eva_gussmann@psych.mpg.de

Table of Contents

1 Supplementary Methods. Background to the intervention development context 3

2 Supplementary Table 1. Synthesis of primary qualitative studies, qualitative metasynthesis, and competence frameworks used for the problem definition and needs analysis of MEBASp (Step 1) 4

3 Supplementary Table 2. Impaired transdiagnostic processes in (acute) psychosis and other disorders (Step 1) 6

4 Supplementary Table 3. Existing mechanism-based therapies for psychosis focusing on impaired metacognitive and cognitive processes (Step 1) 8

5 Supplementary Figure 1. Mechanism-based group therapy concept on the locked acute ward 9

# Supplementary Methods. Background to the intervention development context

1. **Clinic-wide mechanism-based group concept**

Our intervention development project was conducted as a part of the MoMenT ("Modularized and Mechanism-based PsychoTherapy") treatment concept (<https://www.psych.mpg.de/psychotherapie>) at the Max Planck Institute of Psychiatry in Munich, Germany. Within a pilot phase, various brief group therapy modules each focusing on a transdiagnostic change mechanism e.g. emotional regulation or behavioral activation were developed. On admission, the clinical team identifies patient’s preferences and therapy goals and change mechanisms potentially relevant for treatment success and creates an individually targeted therapy plan combining different group modules. For a patient with depression for example, the clinicians’ team might identify repetitive negative thinking as a main causing and maintain mechanism for recurrent depressive episodes. Moreover, the patient reports a severe anhedonia and lack of positive activities. A possible treatment plan could therefore include the group module “mindfulness training” and “Activity group (behavioral activation)”. In the long run, the aim is to personalize treatment and thus optimize patients’ outcomes (1–3). A feasibility study for selected therapy modules is currently in preparation.

1. **“Acute” mechanism-based group concept**

To meet the unique treatment needs of psychiatric inpatients on the acute closed ward, a small subproject group was responsible for creating an adapted "acute" mechanism-based concept (4). The first version of our mechanism-based concept focused on change mechanisms such as distress tolerance, impulsivity reduction, behavioral activation, information processing, and self-management (5). Using these mechanisms, we developed a transdiagnostic Skillstraining, a Resource group, a Psychoeducation group, and a Crisis-competence group (as shown in Supplementary Figure 1). All group interventions were adapted to a crisis-focused setting, designed to be brief, easy to understand, and focused on coping (4). As there were still limited treatment options available for inpatients with acute psychotic symptoms (6), we decided to develop a fifth mechanism-based group (as shown in Supplementary Figure 1). However, since there was little existing evidence for concepts tailored to this patient population and setting (6), the intervention design was accompanied by a rigorous scientific process described in our current work (7).

# Supplementary Table 1. Synthesis of primary qualitative studies, qualitative metasynthesis, and competence frameworks used for the problem definition and needs analysis of MEBASp (Step 1)

| Study | Participants and context | Aim | Results |
| --- | --- | --- | --- |
| Patient experiences of psychiatric inpatient care: a systematic review of qualitative evidence (5) | 11 qualitative studies involving inpatients with different diagnoses from Sweden and the UK | Examine patients’ experience of psychiatric inpatient care | *Themes regarding needs for treatment:* inclusive care, positive relationships with staff, supporting therapeutic environment |
| Acute inpatients’ experiences of stigma from psychosis: A qualitative exploration (8) | 25 acute inpatients from the UK | Examine patients’ subjective experiences of stigma | *Stigmatizing themes:* stigmatising environment, stigmatised person, stigma interactions |
| The therapeutic needs of psychiatric in-patients with psychosis: A qualitative exploration of patient and staff perspectives (9) | 12 acute inpatients and 12 multidisciplinary team members from the UK | Examine patient and staff perspectives on priorities regarding psychological treatment | *Patient themes:* importance to consider social environment and trauma, managing intra- and interpersonal consequences of psychosis, inflexible treatment and dominant pharmacological approach  *Staff themes:* multidisciplinary collaboration, treating complexity and symptom management, restrictive practices preventing quality treatment |
| Sources of Distress in First-Episode Psychosis: A Systematic Review and Qualitative Metasynthesis (10) | 33 qualitative studies involving inpatients and outpatients from Europe, Canada, New Zealand, South Africa, and USA | Increase understanding of self-reported sources of distress | *Intrapersonal distress:* unwanted internal states, conflicts, lost sense of identity, poor health conditions e.g. sleep difficulties  *Interpersonal distress:* traumatic life experiences, distressing contact with health professionals and relatives, stigma |
| Key Components for the Delivery of Cognitive Behavioral Therapies for Psychosis in Acute Psychiatric Inpatient Settings: A Delphi Study of Therapists’ Views (11) | 45 psychological therapists working in psychiatric inpatient care in the UK | Gain consensus on how CBTp should be delivered in acute inpatient settings | *Requirements on CBTp:* normalizing, taking into account patients’ perspectives, reducing distress, recovery-oriented, flexible session content and delivery, adapting to restrictive environment |
| Psychologists’ Perspectives on the implementation of Psychological Therapy for Psychosis in the Acute Psychiatric Inpatient Setting (12) | 12 psychological therapists working in psychiatric inpatient care in the UK | Explore adaptations required to deliver psychological therapies to acute inpatients | *Crisis-focused psychological interventions*: distress reduction, crisis formulation, crisis-focused change mechanisms e.g. mindfulness, normalization and behavioral activation, standalone group interventions  *Working with the wider system:* informed team work, feedback, discharge planning, supporting family system  *Environmental adaptions:* working alongside the medical model, brief interventions, flexible sessions and contents, creative interventions |
| The role of psychology in a multidisciplinary psychiatric inpatient setting: Perspective from the multidisciplinary team (13) | 12 interdisciplinary staff members working in psychiatric inpatient care in the UK | Examine interdisciplinary team’s view on the role of psychology within acute psychiatric settings | *Benefits from psychological treatments:* psychological formulation, delivering group and individual interventions, development of insight and coping strategies, treating interpersonal and intrapersonal difficulties  *Necessary integration into overall concept:* no first line treatment, develop balance to medical model, feedback and supporting the staff team, no clear understanding what psychology does |
| Acute Mental Health Inpatient Competence Framework: Adults and older adults (14) | Expert Reference Group including patients, carers, clinicians and academics in the UK | Provide guidance to staff working in acute inpatient settings | *Psychosocial interventions:* reduce crisis, increase patient’s safety, improve functioning, adapt interventions to crisis setting, provide group-based interventions to offer opportunities for coping, interpersonal skill development, and peer support, provide family interventions |
| A competence framework for psychological interventions with people with psychosis and bipolar disorder (15) | Expert Reference Group including patients, carers, clinicians and academics in the UK | Provide guidance to staff working with people with psychosis and Bipolar Disorder | *Psychosocial interventions for psychosis:* psychoeducation, normalisation, symptom-specific competences focusing on delusions, hallucinations, negative symptoms and trauma, family interventions |

Notes: CBTp = Cognitive Behavioral Therapy for psychosis. Search for qualitative studies involving acute inpatients with psychotic symptoms was conducted using the database Medline and search terms: patient OR inpatient AND psychosis AND mental OR psychiatr* AND hospital OR admission AND qualitative OR interview OR focus group AND experience* (5). All study abstracts were screened to assess the relevance and fit for the current intervention development context.

# Supplementary Table 2. Impaired transdiagnostic processes in (acute) psychosis and other disorders (Step 1)

| **Process domain** | **Impaired process** | **Associated with** | | |
| --- | --- | --- | --- | --- |
|  |  | **Positive symptoms** | **Negative Symptoms** | **Other disorders** |
| Metacognition | Dysfunctional metacognitive beliefs about worry (16) | x AH |  | Anxiety, PTSD, Somatoform, Eating, Mood, Sleep disorders (17) |
|  | Negative beliefs about uncontrollability/  danger/superstition (16) |  |  |  |
|  | Diminished self-reflectivity (18,19) | x AH/D | x | Autism spectrum disorders (20), Psychopathy (21) |
|  | Deficits in self-monitoring (22) | x AH/D | x |  |
|  | Deficits at basic metacognitive levels (23) |  | x | Personality disorders (24) |
|  | Deficits in metacognitive capacity (25) |  | x | OCD (25) |
|  |  |  |  |  |
| Cognition | Higher cognitive confidence (16) | x AH |  |  |
|  | Limited cognitive insight (26) | x AH/D | x | Mood disorders (27) |
|  | Cognitive distortions (reasoning biases such as JTC, attributional biases, biased expectancy, deficits in Theory of mind, confirmation biases) (28) | x D | x | Anxiety, Somatoform, Mood, Eating, Sexual, Impulse control disorders (2) |
|  | Low outcome expectancies (for pleasure/success) (29) |  | x | Panic disorders, Social Phobia, Eating disorders, Somatoform, Substance abuse, Mood disorders, GAD, PTSD (2) |
|  | Low perceived competence (29) |  | x |  |
|  | Impaired source monitoring(30) | x AH |  |  |
|  | High levels of general worry (31) | x D |  | Across all disorders (32) |
|  | Deficits in social cognition (33,34) | x AH/D | x | MDD (35), Autism spectrum disorders (36) |
|  | Cognitive fusion (37,38) | x AH/D |  | OCD (39), Mood, Anxiety disorders (40) |
|  | Dysfunctional coping strategies (41) | x AH/D | x | Across all disorders (42) |
|  |  |  |  |  |
| Memory | Vague and less vivid memory recollection (43) | x AH/D | x | Mood, Anxiety and Eating disorders (2) |
|  | Overconfidence in (false) memory (44,45) | x AH/D | x | Depressive disorders (46) |
|  |  |  |  |  |
| Attention | Selective attention (29) | x AH/D | x | Anxiety, Somatoform, Sexual, Sleep, Mood, Eating, Substance-related disorders (2) |
|  |  |  |  |  |
| Affective | Lower self-esteem (34,47) | x AH/D | x | Mood, Eating, Substance-related disorders (48) |
|  | High emotional reactivity (49) | x AH/D | x | Mood, Anxiety disorders (50) |
|  |  |  |  |  |
| Beliefs | Negative self-concepts |  | x | BPS (34,51), Anxiety (52), Mood disorders (53) |
|  | Defeatist beliefs (54) |  | x | Bipolar (55), Personality disorders, MDD (56) |
|  | Dysfunctional acceptance beliefs (34) |  | x |  |
|  |  |  |  |  |
| Behavior regulation | Incapability to endure ambiguity (57) | x D |  |  |
|  | Less empathic (18) |  | x | Personality (antisocial, Borderline, Narcissist), Autistic spectrum, Mood disorders (58) |
|  | Safety behavior (59) | x AH/D | x | Anxiety, Somatoform, Sleep, Mood, Eating, Substance-related disorders, OCD, PTDS (2) |
|  | Dysfunctional coping strategies (41,60) | x AH/D | x | Across all disorders (42) |
|  |  |  |  |  |
| Social influences | Unsupportive environments (61) | x AH/D | x | Across all disorders (62) |
|  | Trauma and social adversity (63) | x AH |  |  |
|  | Social alienation (64) | x AH/D | x |  |

Key. AH = Auditory Hallucinations; BPS = Borderline personality disorder; D = Delusions; GAD = Generalized anxiety disorder; JTC = Jumping to conclusions; MDD = Major depressive disorder; Mood disorders = Depressive and Bipolar disorders; OCD = Obsessive compulsive disorder; PTSD = Post-traumatic stress disorder

# Supplementary Table 3. Existing mechanism-based therapies for psychosis focusing on impaired metacognitive and cognitive processes (Step 1)

|  | **Target mechanism of change** | **Procedure** | **Evidence-base for psychosis** | **Suitable for**  **acute patients** | **Group format**  **(acute settings)** |
| --- | --- | --- | --- | --- | --- |
| **Metacognitive and cognitive processes** | | | | | |
| **Metacognitive Training (MCT)** | Metacognitive knowledge and awareness, Cognitive restructuring | Raising awareness for cognitive biases and changing the way patients deal with them | Three meta-analyses (65–67) | Current pilot testing of version for acute wards (68) | Available for acute setting |
| **Metacognitive insight and reflection therapy (MERIT)** | Metacognitive awareness | Helping patients to make sense of self and others | Two RCTs (69,70) | Limited, since complex | No group format available |
| **Metacognitive interpersonal therapy for psychosis (MIT-P)** | Metacognitive awareness | Promoting understanding of relationship between emotional distress and symptoms | Three case studies (71–73) | Limited, since complex | No group format available |
| **Cognitive fusion and maladaptive coping strategies** | | | | | |
| **Acceptance and Commitment Therapy (ACT)** | Metacognitive awareness, metacognitive goals and strategies (acceptance/mindfulness/cognitive defusion/values) | Changing the function of thought and voice contents on behavior | One systematic review containing 11 RCTs (74) | Past testing of versions for acute ward (75–77) | Available (not for acute settings) |
| **Mindfulness-based interventions for psychosis** | Metacognitive knowledge, awareness and strategies | Developing mindfulness skills and making sense of crisis | Two feasibility  randomized controlled trials (78,79) | Tested with inpatients, but not explicitly acute | Available (not for acute settings) |
| **Metacognitive Therapy** | Metacognitive knowledge and strategies | Challenging beliefs and training functional coping with thoughts | Two case studies (80,81), one case series (82) and one open trial (83) | Limited, since complex | Available, but only for depression (not for acute settings) |

# Supplementary Figure 1. Mechanism-based group therapy concept on the locked acute ward

Notes: Psychosocial treatment components present the targeted change mechanism with the name of the respective group/treatment module in square brackets. Existing mechanism-based groups on the locked acute ward include a) a transdiagnostic Skillstraining (in total three sessions covering psychoeducation on tension regulation, testing of different stress-tolerance-skills, development of emergency plans and skill chains), b) a transdiagnostic Resource group (in total three sessions covering psychoeducation on depression upward- and downward-spiral, development of positive activities and resources, day and week planning), c) a transdiagnostic Crisis-Competence group (in total four sessions covering crisis formulation, early warning signs and coping strategies, emergency plan and discharge planning, and d) a transdiagnostic Psychoeducation group (in total three sessions covering information on diathesis-stress-model, medication, and treatment options). All groups were adapted from existing group manuals (84–87) to fit the acute inpatient setting. Each group session lasts 50 minutes and takes place weekly. Inpatients are able to participate in two group therapies with the option for individual therapy. The experimental mechanism-based group therapy (in blue) was specifically designed for inpatients with acute psychotic symptoms and takes place twice a week with a total of nine sessions. The ultimate goal of the mechanism-based concept is to individually tailor treatment for acute inpatients by allocating them to the group therapies most likely to target individually relevant change mechanisms and personal preferences.

**References**

1. Hofmann SG, Hayes SC. The Future of Intervention Science: Process-Based Therapy. Clin Psychol Sci. 2019 Jan;7(1):37–50.

2. Herpertz S, Schramm E, Deisenhofer AK, editors. Modulare Psychotherapie: ein Mechanismus-basiertes, personalisiertes Vorgehen. Stuttgart: Schattauer; 2022. 153 p.

3. Elsaesser M, Herpertz S, Piosczyk H, Jenkner C, Hautzinger M, Schramm E. Modular-based psychotherapy (MoBa) versus cognitive–behavioural therapy (CBT) for patients with depression, comorbidities and a history of childhood maltreatment: study protocol for a randomised controlled feasibility trial. BMJ Open. 2022 Jul;12(7):e057672.

4. Bowers L, Chaplin R, Quirk A, Lelliott P. A conceptual model of the aims and functions of acute inpatient psychiatry. J Ment Health. 2009 Jan;18(4):316–25.

5. Wood L, Alsawy S. Patient experiences of psychiatric inpatient care: a systematic review of qualitative evidence. J Psychiatr Intensive Care. 2016 Apr 1;12(1):35–43.

6. Barnicot K, Michael C, Trione E, Lang S, Saunders T, Sharp M, et al. Psychological interventions for acute psychiatric inpatients with schizophrenia-spectrum disorders: A systematic review and meta-analysis. Clin Psychol Rev. 2020 Dec;82:101929.

7. Bleijenberg N, de Man-van Ginkel JM, Trappenburg JCA, Ettema RGA, Sino CG, Heim N, et al. Increasing value and reducing waste by optimizing the development of complex interventions: Enriching the development phase of the Medical Research Council (MRC) Framework. Int J Nurs Stud. 2018 Mar;79:86–93.

8. Wood L, Byrne R, Enache G, Morrison AP. Acute inpatients’ experiences of stigma from psychosis: A qualitative exploration. Stigma Health. 2018 Feb;3(1):1–8.

9. Wood L, Williams C, Billings J, Johnson S. The therapeutic needs of psychiatric in-patients with psychosis: A qualitative exploration of patient and staff perspectives. BJPsych Open. 2019 May;5(3):e45.

10. Griffiths R, Mansell W, Edge D, Tai S. Sources of Distress in First-Episode Psychosis: A Systematic Review and Qualitative Metasynthesis. Qual Health Res. 2019 Jan;29(1):107–23.

11. Wood L, Jacobsen P, Ovin F, Morrison AP. Key Components for the Delivery of Cognitive Behavioral Therapies for Psychosis in Acute Psychiatric Inpatient Settings: A Delphi Study of Therapists’ Views. Schizophr Bull Open. 2022 Jan 1;3(1):sgac005.

12. Wood L, Williams C, Billings J, Johnson S. Psychologists’ Perspectives on the implementation of Psychological Therapy for Psychosis in the Acute Psychiatric Inpatient Setting. Qual Health Res. 2019 Dec;29(14):2048–56.

13. Wood L, Williams C, Billings J, Johnson S. The role of psychology in a multidisciplinary psychiatric inpatient setting: Perspective from the multidisciplinary team. Psychol Psychother Theory Res Pract. 2019 Dec;92(4):554–64.

14. Wood L, Williams C. Acute Mental Health Inpatient Competence Framework: Adults and older adults [Internet]. University College London; 2022. Available from: https://www.ucl.ac.uk/pals/sites/pals/files/background_document_competency_framework_final_draft.pdf

15. Roth A, Pilling S. A competence framework for psychological interventions with people with psychosis and bipolar disorder [Internet]. University College London; 2012. Available from: https://www.ucl.ac.uk/clinicalpsychology//CORE/Docs/Working%20with%20Psychosis%20and%20Bipolar%20Disorder%20background%20document%20web%20version.pdf

16. Morrison AP, Wells A. A comparison of metacognitions in patients with hallucinations, delusions, panic disorder, and non-patient controls. Behav Res Ther. 2003 Feb;41(2):251–6.

17. Harvey AG. A cognitive model of insomnia. Behav Res Ther. 2002 Aug;40(8):869–93.

18. Tas C, Brown EC, Aydemir O, Brüne M, Lysaker PH. Metacognition in psychosis: Comparison of schizophrenia with bipolar disorder. Psychiatry Res. 2014 Nov;219(3):464–9.

19. García-Mieres H, De Jesús-Romero R, Ochoa S, Feixas G. Beyond the cognitive insight paradox: Self-reflectivity moderates the relationship between depressive symptoms and general psychological distress in psychosis. Schizophr Res. 2020 Aug;222:297–303.

20. Lombardo MV, Chakrabarti B, Bullmore ET, Sadek SA, Pasco G, Wheelwright SJ, et al. Atypical neural self-representation in autism. Brain. 2010 Feb 1;133(2):611–24.

21. Koenigs M, Huey ED, Calamia M, Raymont V, Tranel D, Grafman J. Distinct Regions of Prefrontal Cortex Mediate Resistance and Vulnerability to Depression. J Neurosci. 2008 Nov 19;28(47):12341–8.

22. Frith C. The neural basis of hallucinations and delusions. C R Biol. 2005 Feb;328(2):169–75.

23. Lysaker PH, Kukla M, Dubreucq J, Gumley A, McLeod H, Vohs JL, et al. Metacognitive deficits predict future levels of negative symptoms in schizophrenia controlling for neurocognition, affect recognition, and self-expectation of goal attainment. Schizophr Res. 2015 Oct;168(1–2):267–72.

24. Semerari A, Colle L, Pellecchia G, Buccione I, Carcione A, Dimaggio G, et al. Metacognitive Dysfunctions in Personality Disorders: Correlations With Disorder Severity and Personality Styles. J Personal Disord. 2014 Dec;28(6):751–66.

25. García-Montes JM, Pérez-Álvarez M, Soto Balbuena C, Perona Garcelán S, Cangas AJ. Metacognitions in patients with hallucinations and obsessive-compulsive disorder: The superstition factor. Behav Res Ther. 2006 Aug;44(8):1091–104.

26. Nair A, Palmer EC, Aleman A, David AS. Relationship between cognition, clinical and cognitive insight in psychotic disorders: A review and meta-analysis. Schizophr Res. 2014 Jan;152(1):191–200.

27. Colis MJ, Steer RA, Beck AT. Cognitive Insight in Inpatients with Psychotic, Bipolar, and Major Depressive Disorders. J Psychopathol Behav Assess. 2006 Nov 6;28(4):242–9.

28. Moritz S, Woodward TS. Metacognitive training in schizophrenia: from basic research to knowledge translation and intervention. Curr Opin Psychiatry. 2007 Nov;20(6):619–25.

29. Rector NA, Beck AT, Stolar N. The Negative Symptoms of Schizophrenia: A Cognitive Perspective. Can J Psychiatry. 2005 Apr;50(5):247–57.

30. Keefe RSE, Arnold MC, Bayen UJ, Harvey PD. Source monitoring deficits in patients with schizophrenia; a multinomial modelling analysis. Psychol Med. 1999 Jul;29(4):903–14.

31. Freeman D, Garety PA. Worry, Worry processes and dimensions of delusions: An exploratory investigation of a role for anxiety porcesses in the maintenance of delusional distress. Behav Cogn Psychother. 1999 Jan;27(1):47–62.

32. Ehring T. Thinking too much: rumination and psychopathology. World Psychiatry. 2021 Oct;20(3):441–2.

33. Bliksted V, Videbech P, Fagerlund B, Frith C. The effect of positive symptoms on social cognition in first-episode schizophrenia is modified by the presence of negative symptoms. Neuropsychology. 2017 Feb;31(2):209–19.

34. Lincoln TM, Mehl S, Kesting ML, Rief W. Negative Symptoms and Social Cognition: Identifying Targets for Psychological Interventions. Schizophr Bull. 2011 Sep 1;37(suppl 2):S23–32.

35. Weightman MJ, Air TM, Baune BT. A Review of the Role of Social Cognition in Major Depressive Disorder. Front Psychiatry [Internet]. 2014 Dec 11 [cited 2022 Jul 17];5. Available from: http://journal.frontiersin.org/article/10.3389/fpsyt.2014.00179/abstract

36. Derntl B, Habel U. Deficits in social cognition: a marker for psychiatric disorders? Eur Arch Psychiatry Clin Neurosci. 2011 Nov;261(S2):145–9.

37. Newman-Taylor K, Richardson T, Lees R, Petrilli K, Bolderston H, Hindocha C, et al. Cognitive fusion as a candidate psychological vulnerability factor for psychosis: An experimental study of acute ∆9-tetrahydrocannabinol (THC) intoxication. Psychosis. 2021 Apr 3;13(2):167–74.

38. Moran O, Larsson A, McHugh L. Investigating cognitive fusion, mindfulness and experiential avoidance in relation to psychosis-like symptoms in the general population. J Context Behav Sci. 2021 Jul;21:136–43.

39. Xiong A, Lai X, Wu S, Yuan X, Tang J, Chen J, et al. Relationship Between Cognitive Fusion, Experiential Avoidance, and Obsessive–Compulsive Symptoms in Patients With Obsessive–Compulsive Disorder. Front Psychol. 2021 Apr 12;12:655154.

40. Bardeen JR, Fergus TA. The interactive effect of cognitive fusion and experiential avoidance on anxiety, depression, stress and posttraumatic stress symptoms. J Context Behav Sci. 2016 Jan;5(1):1–6.

41. Moritz S, Lüdtke T, Westermann S, Hermeneit J, Watroba J, Lincoln TM. Dysfunctional coping with stress in psychosis. An investigation with the Maladaptive and Adaptive Coping Styles (MAX) questionnaire. Schizophr Res. 2016 Aug;175(1–3):129–35.

42. Moritz S, Jahns AK, Schröder J, Berger T, Lincoln TM, Klein JP, et al. More adaptive versus less maladaptive coping: What is more predictive of symptom severity? Development of a new scale to investigate coping profiles across different psychopathological syndromes. J Affect Disord. 2016 Feb;191:300–7.

43. Sheffield JM, Karcher NR, Barch DM. Cognitive Deficits in Psychotic Disorders: A Lifespan Perspective. Neuropsychol Rev. 2018 Dec;28(4):509–33.

44. Kircher TTJ, Koch K, Stottmeister F, Durst V. Metacognition and Reflexivity in Patients with Schizophrenia. Psychopathology. 2007;40(4):254–60.

45. Moritz S, Woodward TS. Metacognitive control over false memories: A key determinant of delusional thinking. Curr Psychiatry Rep. 2006 May;8(3):184–90.

46. Moritz S, Gläscher J, Brassen S. Investigation of mood-congruent false and true memory recognition in depression: Research Article: False Memory in Depression. Depress Anxiety. 2005;21(1):9–17.

47. Haug E, Øie MG, Andreassen OA, Bratlien U, Romm KL, Møller P, et al. The Association between Anomalous Self-experiences, Self-esteem and Depressive Symptoms in First Episode Schizophrenia. Front Hum Neurosci [Internet]. 2016 Nov 7 [cited 2022 Jun 25];10. Available from: http://journal.frontiersin.org/article/10.3389/fnhum.2016.00557/full

48. Silverstone PH, Salsali M. [No title found]. Ann Gen Hosp Psychiatry. 2003;2(1):2.

49. Myin-Germeys I, Krabbendam L, Delespaul P a. EG, van Os J. Sex differences in emotional reactivity to daily life stress in psychosis. J Clin Psychiatry. 2004 Jun;65(6):805–9.

50. Pine DS, Cohen P, Brook JS. Emotional Reactivity and Risk for Psychopathology Among Adolescents. CNS Spectr. 2001 Jan;6(1):27–35.

51. Winter D, Bohus M, Lis S. Understanding Negative Self-Evaluations in Borderline Personality Disorder—a Review of Self-Related Cognitions, Emotions, and Motives. Curr Psychiatry Rep. 2017 Mar;19(3):17.

52. Krull T, Leibing E, Pöhlmann K, Leichsenring F, Salzer S. Das Selbstkonzept von Patienten mit sozialer Angststörung:Ausprägung und Veränderung durch Psychotherapie. Z Für Psychosom Med Psychother. 2014 May 1;60(2):162–76.

53. Reed-Fitzke K. The Role of Self-Concepts in Emerging Adult Depression: A Systematic Research Synthesis. J Adult Dev. 2020 Mar;27(1):36–48.

54. Grant PM, Beck AT. Defeatist Beliefs as a Mediator of Cognitive Impairment, Negative Symptoms, and Functioning in Schizophrenia. Schizophr Bull. 2009 Jul 1;35(4):798–806.

55. Reilly-Harrington NA, Miklowitz DJ, Otto MW, Frank E, Wisniewski SR, Thase ME, et al. Dysfunctional Attitudes, Attributional Styles, and Phase of Illness in Bipolar Disorder. Cogn Ther Res. 2010 Feb;34(1):24–34.

56. Farabaugh A, Mischoulon D, Schwartz F, Pender M, Fava M, Alpert J. Dysfunctional attitudes and personality disorder comorbidity during long-term treatment of MDD. Depress Anxiety. 2007;24(6):433–9.

57. Colbert SM, Peters ER, Garety PA. Need for closure and anxiety in delusions: A longitudinal investigation in early psychosis. Behav Res Ther. 2006 Oct;44(10):1385–96.

58. Decety J, Moriguchi Y. The empathic brain and its dysfunction in psychiatric populations: implications for intervention across different clinical conditions. Biopsychosoc Med. 2007;1(1):22.

59. Gaynor K, Ward T, Garety P, Peters E. The role of safety-seeking behaviours in maintaining threat appraisals in psychosis. Behav Res Ther. 2013 Feb;51(2):75–81.

60. Freeman D. Persecutory delusions: a cognitive perspective on understanding and treatment. Lancet Psychiatry. 2016 Jul;3(7):685–92.

61. Norman RMG, Malla AK, Manchanda R, Townsend L. Premorbid adjustment in first episode schizophrenia and schizoaffective disorders: a comparison of social and academic domains. Acta Psychiatr Scand. 2005 Jul;112(1):30–9.

62. Alegría M, NeMoyer A, Falgàs Bagué I, Wang Y, Alvarez K. Social Determinants of Mental Health: Where We Are and Where We Need to Go. Curr Psychiatry Rep. 2018 Nov;20(11):95.

63. Read J, Os J, Morrison AP, Ross CA. Childhood trauma, psychosis and schizophrenia: a literature review with theoretical and clinical implications. Acta Psychiatr Scand. 2005 Nov;112(5):330–50.

64. Leonhardt BL, Huling K, Hamm JA, Roe D, Hasson-Ohayon I, McLeod HJ, et al. Recovery and serious mental illness: a review of current clinical and research paradigms and future directions. Expert Rev Neurother. 2017 Nov 2;17(11):1117–30.

65. Penney D, Sauvé G, Mendelson D, Thibaudeau É, Moritz S, Lepage M. Immediate and Sustained Outcomes and Moderators Associated With Metacognitive Training for Psychosis: A Systematic Review and Meta-analysis. JAMA Psychiatry. 2022 May 1;79(5):417.

66. Liu Y, Tang C, Hung T, Tsai P, Lin M. The Efficacy of Metacognitive Training for Delusions in Patients With Schizophrenia: A Meta‐Analysis of Randomized Controlled Trials Informs Evidence‐Based Practice. Worldviews Evid Based Nurs. 2018 Apr;15(2):130–9.

67. Eichner C, Berna F. Acceptance and Efficacy of Metacognitive Training (MCT) on Positive Symptoms and Delusions in Patients With Schizophrenia: A Meta-analysis Taking Into Account Important Moderators. Schizophr Bull. 2016 Jul;42(4):952–62.

68. Fischer R, Scheunemann J, Bohlender A, Duletzki P, Nagel M, Moritz S. ‘You are trying to teach us to think more slowly!’: Adapting Metacognitive Training for the acute care setting—A case report. Clin Psychol Psychother. 2022 May 25;cpp.2755.

69. Vohs JL, Leonhardt BL, James AV, Francis MM, Breier A, Mehdiyoun N, et al. Metacognitive Reflection and Insight Therapy for Early Psychosis: A preliminary study of a novel integrative psychotherapy. Schizophr Res. 2018 May;195:428–33.

70. de Jong S, van Donkersgoed RJM, Timmerman ME, aan het Rot M, Wunderink L, Arends J, et al. Metacognitive reflection and insight therapy (MERIT) for patients with schizophrenia. Psychol Med. 2019 Jan;49(2):303–13.

71. Salvatore G, Procacci M, Popolo R, Nicolò G, Carcione A, Semerari A, et al. Adapted Metacognitive Interpersonal Therapy for Improving Adherence to Intersubjective Contexts in a Person With Schizophrenia. Clin Case Stud. 2009 Dec;8(6):473–88.

72. Salvatore G, Ottavi P, Popolo R, Dimaggio G. Metacognitive Interpersonal Therapy for Treating Auditory Verbal Hallucinations in First-onset Schizophrenia. J Contemp Psychother. 2016 Dec;46(4):235–43.

73. Salvatore G, Buonocore L, Ottavi P, Popolo R, Dimaggio G. Metacognitive Interpersonal Therapy for Treating Persecutory Delusions in Schizophrenia. Am J Psychother. 2018 Dec;71(4):164–74.

74. Yıldız E. The effects of acceptance and commitment therapy in psychosis treatment: A systematic review of randomized controlled trials. Perspect Psychiatr Care. 2020 Jan;56(1):149–67.

75. Bach P, Gaudiano BA, Hayes SC, Herbert JD. Acceptance and commitment therapy for psychosis: intent to treat, hospitalization outcome and mediation by believability. Psychosis. 2013 Jun;5(2):166–74.

76. Gaudiano BA, Herbert JD. Acute treatment of inpatients with psychotic symptoms using Acceptance and Commitment Therapy: Pilot results. Behav Res Ther. 2006 Mar;44(3):415–37.

77. Tyrberg MJ, Carlbring P, Lundgren T. Brief acceptance and commitment therapy for psychotic inpatients: A randomized controlled feasibility trial in Sweden. Nord Psychol. 2017 Apr 3;69(2):110–25.

78. Böge K, Hahne I, Bergmann N, Wingenfeld K, Zierhut M, Thomas N, et al. Mindfulness-based group therapy for in-patients with schizophrenia spectrum disorders – Feasibility, acceptability, and preliminary outcomes of a rater-blinded randomized controlled trial. Schizophr Res. 2021 Feb;228:134–44.

79. Jacobsen P, Hodkinson K, Peters E, Chadwick P. A systematic scoping review of psychological therapies for psychosis within acute psychiatric in-patient settings. Br J Psychiatry. 2018 Aug;213(2):490–7.

80. Hutton P, Morrison AP, Taylor H. Brief Cognitive Behavioural Therapy for Hallucinations: Can it Help People Who Decide Not to Take Antipsychotic Medication? A Case Report. Behav Cogn Psychother. 2012 Jan;40(1):111–6.

81. Valmaggia LR, Bouman TK, Schuurman L. Attention Training With Auditory Hallucinations: A Case Study. Cogn Behav Pract. 2007 May;14(2):127–33.

82. Hutton P, Morrison AP, Wardle M, Wells A. Metacognitive Therapy in Treatment-Resistant Psychosis: A Multiple-Baseline Study. Behav Cogn Psychother. 2014 Mar;42(2):166–85.

83. Morrison AP, Pyle M, Chapman N, French P, Parker SK, Wells A. Metacognitive therapy in people with a schizophrenia spectrum diagnosis and medication resistant symptoms: A feasibility study. J Behav Ther Exp Psychiatry. 2014 Jun;45(2):280–4.

84. Bäuml J, editor. Handbuch der Psychoedukation: für Psychiatrie, Psychotherapie und Psychosomatische Medizin; mit ... 61 Tabellen. Stuttgart: Schattauer; 2016. 640 p.

85. Bohus M, Wolf-Arehult M. Interaktives Skillstraining für Borderline-Patienten: das Therapeutenmanual: mit 158 Info- und Arbeitsblättern. 2., aktualisierte und erweiterte Auflage. Stuttgart: Schattauer; 2016. 409 p.

86. Hofheinz C, Heidenreich T, Michalak J. Werteorientierte Verhaltensaktivierung bei depressiven Störungen: Therapiemanual: mit E-Book inside und Arbeitsmaterial. 1. Auflage. Weinheim Basel: Beltz; 2017. 198 p.

87. Lindenmeyer J, editor. Alkoholabhängigkeit, Angststörungen, Arbeitstherapie, Genusstraining, Männliche Sexualität und Partnerschaft, Nachsorge, Partnerseminar, Pathologischer PC- und Internetgebrauch, Pathologisches Glücksspiel, Raucherentwöhnung, Stress am Arbeitsplatz: mit E-Book inside und Arbeitsmaterial. 3., überarbeitete und erweiterte Auflage. Weinheim Basel: Beltz; 2021. 319 p. (Therapie-Tools Gruppentherapie / Johannes Lindenmeyer (Hrsg.)).
